# Unfavorable effects of history of volume overload and late referral to a nephrologist on mortality in patients initiating dialysis: a multicenter prospective cohort study in Japan

**DOI:** 10.1186/s12882-018-0859-8

**Published:** 2018-03-14

**Authors:** Masaki Okazaki, Daijo Inaguma, Takahiro Imaizumi, Akiko Kada, Takaaki Yaomura, Naotake Tsuboi, Shoichi Maruyama

**Affiliations:** 1 Department of Nephrology, Nagoya University Graduate School of Medicine, 65 Tsurumai-cho, Showa-ku, Nagoya, 466-8550 Japan; 2 Department of Nephrology, Fujita Health University School of Medicine, Toyoake, Japan; 3 Department of Clinical Trials and Research, Clinical Research Center, National Hospital Organization Nagoya Medical Center, Nagoya, Japan; 4 Department of Nephrology, National Hospital Organization Nagoya Medical Center, Nagoya, Japan

**Keywords:** Predialysis nephrologist care, Early versus late referral, Hemodialysis initiation

## Abstract

**Background:**

Patients with late referral and positive history of volume overload may have a poor prognosis after initiating dialysis due to insufficient and/or inadequate management of complications of renal failure and the lack of better dialysis preparation. Little is known about the influence of the relationship between history of volume overload and late referral on prognosis.

**Methods:**

We analyzed 1475 patients who had initiated dialysis for the first time from October 2011 to September 2013. late referral was defined as referral to a nephrologist < 3 months before dialysis initiation. The major outcomes were all-cause death and deaths due to cardiovascular diseases (CVD). The impact of late referral and history of volume overload on all-cause mortality was assessed by Cox proportional hazards models.

**Results:**

Among 1475 patients, the mean patient age was 67.5 years. During the median follow-up of 2.2 years, 260 deaths occurred; 99 were due to CVD. Cox proportional hazards models demonstrated that late referral (adjusted hazard ratio [HR], 1.35; 95% confidence interval [CI], 1.00–1.82) and history of volume overload (adjusted HR, 1.39; 95% CI, 1.06–1.81) were risk factors for all-cause mortality. Furthermore, late referral coexisting was associated with a history of volume overload increased mortality (adjusted HR, 2.10; 95% CI, 1.39–3.16 versus absence of late referral without history of volume overload) after adjusting for age, sex, diabetes, atherosclerotic disease, and laboratory values.

**Conclusions:**

Both late referral and history of volume overload were associated with increased risks of all-cause mortality.

**Trial registration:**

University Hospital Medical Information Network (UMIN000007096). Registered 18 January 2012, retrospectively registered.

https://upload.umin.ac.jp/cgi-open-bin/ctr_e/ctr_view.cgi?recptno=R000008349.

**Electronic supplementary material:**

The online version of this article (10.1186/s12882-018-0859-8) contains supplementary material, which is available to authorized users.

## Background

Currently in Japan, it is estimated that over 12 million adults have chronic kidney disease (CKD). In patients with CKD progressing to end-stage renal disease (ESRD), timely referral to a nephrologist and adequate nephrologist care are important for managing several risk factors associated with adverse outcomes. The major benefits of nephrology care in CKD patients include a) identification of reversible causes of renal failure and slowing the rate of progression to ESRD; b) management of complications of renal failure, including volume overload which can lead to left ventricular hypertrophy, mineral and bone disorders associated with cardiovascular disease (CVD), hypertension, and anemia; and c) better preparation for renal replacement therapy (RRT) and placement of dialysis access [1–3]. Therefore, late referral to a nephrologist could be associated with several unfavorable outcomes including impaired opportunity for choice of dialysis modality [4, 5], inadequate preparation of vascular access [6–8], and a higher mortality rate after starting maintenance dialysis [1, 9]. However, in a previous study of elderly patients aged over 67 years, despite the significant trends both in the decrease in the proportion of patients who had consulted with a nephrologist less than 3 months before initiating dialysis and in the improvement of anemia due to increased use of erythropoiesis-stimulating agents, the increase in survival rates after dialysis initiation were surprisingly poor [2]. Considering the leading cause of death for patients with ESRD is CVD [10], further studies are needed to investigate the risk factors associated with mortality after dialysis in patients with late referral.

Patients with CKD are prone to volume overload due to their poor ability to respond to the rapid uptake of sodium, even before the onset of ESRD. Recent studies have shown that volume overload, whether determined clinically or using bioelectric impedance method, is associated with a rapid decline in the estimated glomerular filtration rate (eGFR) and increased mortality risk in advanced CKD patients [11–13]. Thus, if advanced CKD patients with volume overload are delayed referral to a nephrologist, the preparation period before initiating dialysis may be shorter than expected. However, in a prior study of patients with stage 3–5 CKD using the bioimpedance method, only about half were euvolemic, and about 20% of patients with volume overload had no clinically detectable edema [14]. Therefore, even the past clinical history of volume overload during the period of progression of CKD to ESRD may suggest a potential predisposition of volume overload. However, to our knowledge, few studies have evaluated how the association between late referral and history of volume overload affects mortality in patients initiating dialysis. Additionally, large prospective studies that evaluate the relationship between the effects of late referral and multiple clinical complications associated with CVD are lacking [1].

In the present study, we evaluated the effects and the relationships between late referral and clinical history of volume overload on all-cause mortality and CVD-related mortality among patients newly initiated to dialysis registered with the multicenter prospective study, the Aichi Cohort Study of Prognosis in Patients Newly Initiated into Dialysis (AICOPP).

## Methods

### Study population

**Fig. 1:**
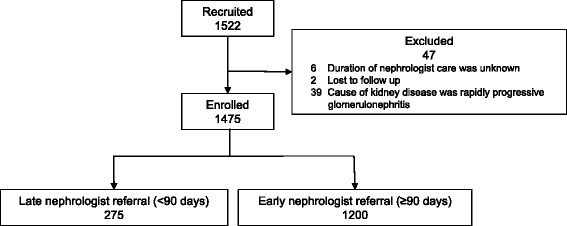
Flow diagram illustrating patient enrollment for the present study

This multicenter prospective cohort study included 1522 patients with ESRD. These patients were recruited from 17 participating clinical centers from October 2011 to September 2013. The exclusion criteria were as follows: (i) age < 20 years, (ii) death during hospitalization, and (iii) patient refusal of registration. Of the 1522 recruited patients, we excluded 8 patients whose period of nephrology care before initiating dialysis or prognoses was unknown and 39 patients whose underlying kidney disease was rapidly progressive glomerulonephritis. Our study enrolled 1475 patients whose period of nephrology care was derived from medical charts (Fig. 1). The AICOPP study originally aimed to examine the effects of predialysis nephrology care and conditions of comorbidities on mortality after initiating maintenance dialysis. AICOPP participants were followed through direct medical chart audit until death or kidney transplant. The last date of follow-up was March 31, 2015. Outcomes were determined by surveying medical records of the AICOPP group institutions or by sending letters to facilities where patients had been transferred for maintenance dialysis. These letters included a questionnaire regarding the outcomes, including cause of death and date of onset. During the observation period, patients who received kidney transplantation (*n* = 23) or were lost to follow-up (n = 2) were censored and were included in the analyses. The cause of death was confirmed in the chart by using a death certificate together with the local investigator when death occurred at the AICOPP group institutions. For the patients who were transferred to another dialysis facility, the cause of death was judged by the attending dialysis physician based on the information obtained from the hospital where the patients died or from the primary cause of admission The AICOPP study was conducted by using the “ethical guidelines for clinical research” of the Japanese Ministry of Health, Labor, and Welfare (created on July 30, 2003; fully revised on July 31, 2008), and was registered at the University Hospital Medical Information Network on January 18, 2012 (ID 000007096). The study protocol was approved by the institutional review board at each participating institution. Informed consent was obtained from all recruited individual participants included in the AICOPP. All clinical investigations were conducted in accordance with the ethical principles of the Declaration of Helsinki.

### Data collection and outcome

Detailed demographic information, past medical history and comorbidities, date of first referral to a nephrologist, laboratory values, initial dialysis modality, initial vascular access, oral medications, and clinical conditions at initiation of dialysis initiation was obtained from the medical records at each dialysis center [15]. All the enrolled patients started maintenance dialysis at the center. Local investigators from each institution obtained the information on comorbidities and past medical history from the chart, including clinical information from the referring primary physicians. History on the cardiovascular system or the metabolic system was obtained. The baseline was defined as the time at which maintenance dialysis was initiated. Diabetes mellitus was defined as a fasting blood glucose level ≥ 126 mg/dL, random blood glucose level ≥ 200 mg/dL, HbA1c (National Glycohemoglobin Standardization Program) level ≥ 6.5%, use of insulin, or use of oral hypoglycemic agents. Laboratory tests were performed using blood samples obtained before the first dialysis session. Predialysis body weight and predialysis blood pressure were also measured just prior to the first dialysis session. Information on oral medication was obtained from the medical records. Oral medication refers to the drugs used at the time of dialysis initiation. The eGFR was calculated using the estimation formula of the Japanese Society of Nephrology [16]. The cardiac ejection fraction was investigated for 1216 of the 1475 (82.4%) patients enrolled who had undergone echocardiography during the 3 months before hospitalization and initiation of maintenance dialysis.

The primary endpoint of our study was all-cause death. We also investigated the etiology of each death, and in particular of CVD-related deaths.

### Definition

We defined late referral as referral to a nephrologist < 3 months between the first nephrology evaluation and dialysis initiation (Fig. 1). A period of 3 months before the initiation of dialysis is the most widely accepted threshold for discriminating between ER and late referral, because it is generally considered the minimum essential time required to prepare for RRT (e.g., creation of arteriovenous fistula) [17]. The clinical history of volume overload was evaluated by investigators at each dialysis institution. The history of volume overload was defined according to the following objective clinical findings obtained at least once during the period from CKD to ESRD: pulmonary congestion or pleural fluid seen on plain chest radiography and physical findings of lower extremity edema. Any incidence of volume overload that occurred for the first time during the phase of dialysis induction was excluded from the definition of the history of volume overload. The history of volume overload was also distinguished from heart failure symptoms at the start of maintenance dialysis or past history of hospitalization for heart failure. The following findings were considered heart failure symptoms: (1) dyspnea or orthopnea with hypoxemia; (2) weight gain or jugular venous distension [18]. Coronary heart disease was defined as an angiographically confirmed occlusion or stenosis of one or more coronary arteries, causing myocardial infarction or angina pectoris [19]. Peripheral artery disease was defined as a disease that required admission for revascularization or amputation surgery. In the present study, we also defined atherosclerotic disease as coronary heart disease and/or peripheral artery disease and/or cerebrovascular disease.

### Statistical analysis

Demographic and clinical characteristics and laboratory profile values are expressed as mean ± standard deviation or median for continuous variables and percentage of the total for categorical variables. The *t* test was used for continuous variables with approximately normal distributions, and the chi-square test for categorical data, as appropriate.

First, we constructed four Cox proportional hazards models to determine the risk of all-cause mortality associated with late referral and clinical characteristics. Variables incorporated into the Cox proportional hazards models were selected from among those significant in univariate analysis, or clinically important variables. Model 1 was adjusted for age, sex and history of volume overload. Model 2 was adjusted for the variables in model 1 with the addition of history of malignancy and comorbidities including diabetes mellitus and atherosclerotic disease. Model 3 was adjusted for the variables in model 2 with the addition of serum albumin and phosphate. Model 4 was adjusted for the variables in model 3 with the addition of hemoglobin to evaluate the relationship between the effects of late referral on overall death and the pre-ESRD management of complications from renal failure. The association between late referral and CVD-related death was assessed using a competing risk analysis. CVD-related death was the event of interest, and non-CVD-related death was considered as the competing event. Kidney transplantation or loss to follow-up were considered to be censoring events. A cumulative incidence function was created using R, version 3.4.3. Secondly, the patients were stratified into four groups according to the timing of referral to a nephrologist, the presence or absence of late referral, and the history of volume overload, with or without a history of volume overload. We presented all-cause mortality with Kaplan-Meier curves for these four groups. Third, to evaluate the relationship between late referral and history of volume overload on all-cause mortality, we analyzed the four stratified groups as categorical variables using Cox proportional hazards models adjusting for age, sex, comorbidities, and laboratory values as covariates. The proportional hazards assumption for covariates was tested using scaled Schoenfeld residuals. We also examined the interaction between late referral and history of volume overload to evaluate the effect modification on overall mortality. Statistical analyses were performed with Stata SE version 15.0 (StataCorp, College Station, TX). A *P* value of < 0.05 was considered statistically significant.

## Results

### Patient characteristics

The characteristics of patients with late referral and without late referral are shown in Table 1. Among the 1475 patients enrolled, 275 (18.6%) were referred late to a nephrologist. The mean (± standard deviation) age at starting dialysis was 67.5 ± 13.1 years, and the percentage of male patients was 68.1%. We identified a cohort of patients receiving maintenance dialysis with similar age and sex distribution to that reported in the Japanese Society of Dialysis Therapy registry data [20, 21]. Patients with late referral showed significantly higher proportion of the following: clinical history of volume overload, peripheral artery disease, and dementia. The albumin and hemoglobin levels were lower, whereas eGFR and phosphate concentrations were higher in late referral patients. The prevalence of peritoneal dialysis was lower in late referral. In terms of initial dialysis access applied, the placement of arteriovenous fistula was less common and the use of central venous catheter was more common in the late referral group than in the absence of late referral group. The late referral group showed significantly lower administration of angiotensin-receptor blockers, angiotensin-converting enzyme inhibitors, β-blockers, calcium antagonists, statins, loop diuretics, vitamin D receptor activator, and antiplatelet agents than the absence of late referral group. Regarding the conditions occurring at the first sessions of maintenance dialysis, the proportion of late referral group presenting heart failure symptoms was high.

**Table 1:** Baseline characteristics of patients at dialysis initiation by referral timing to a nephrologist

	Total (*n* = 1475)	Late referral (*n* = 275)	Without late referral (*n* = 1200)	*P* value (Late vs. Non late)
Characteristics				
Age (years), mean ± SD	67.5 ± 13.1	67.0 ± 13.7	67.6 ± 12.9	0.6
Male sex, *n* (%)	1005 (68.1)	183 (66.5)	822 (68.5)	0.5
Medical history, *n* (%)				
Volume overload	381 (25.8)	97 (35.3)	284 (23.7)	< 0.001
Admission for heart failure	308 (20.9)	64 (23.3)	244 (20.3)	0.3
Amputation	24 (1.6)	6 (2.2)	18 (1.5)	0.4
Malignancy	158 (10.7)	20 (7.3)	138 (11.5)	0.04
Comorbidity, *n* (%)				
Diabetes mellitus	767 (52.0)	129 (46.9)	638 (53.2)	0.06
Atherosclerotic disease	405 (27.5)	71 (25.8)	334 (27.8)	0.5
Coronary heart disease	251 (17.1)	39 (14.2)	212 (17.7)	0.2
Valvular heart disease	99 (6.7)	13 (4.7)	86 (7.2)	0.1
Aortic disease	83 (5.6)	20 (7.3)	63 (5.3)	0.2
Peripheral artery disease	76 (5.2)	21 (7.6)	55 (4.6)	0.04
Cerebrovascular disease	136 (9.2)	24 (8.7)	112 (9.3)	0.8
Chronic obstructive pulmonary disease	50 (3.4)	11 (4.0)	39 (3.3)	0.5
Peptic ulcer disease	51 (3.5)	14 (5.1)	37 (3.1)	0.1
Liver disease	67 (4.5)	12 (4.4)	55 (4.6)	0.9
Dementia	148 (10.0)	37 (13.5)	112 (9.3)	0.04
Laboratory values				
Hemoglobin (g/dL), mean ± SD	9.4 ± 1.5	8.8 ± 1.8	9.5 ± 1.4	< 0.001
Serum albumin (g/dL), mean ± SD	3.2 ± 0.6	3.0 ± 0.6	3.2 ± 0.6	< 0.001
eGFR (mL/min per 1.73 m^2^), mean ± SD	5.5 ± 2.2	5.9 ± 3.3	5.4 ± 1.9	0.01
Potassium (mEq/L), mean ± SD	4.5 ± 0.8	4.6 ± 1.0	4.5 ± 0.8	0.3
Adjusted calcium (mg/dL), mean ± SD	8.6 ± 1.1	8.6 ± 1.2	8.6 ± 1.0	0.5
Phosphate (mg/dL), mean ± SD	6.4 ± 1.9	6.7 ± 2.4	6.3 ± 1.7	< 0.001
Charlson comorbidity index	4.8 ± 1.8	5.0 ± 2.0	4.8 ± 1.8	0.2
Dialysis modality, *n* (%)				
Peritoneal dialysis	104 (7.1)	11 (4.0)	93 (7.8)	0.03
Initial dialysis access, *n* (%)				< 0.001
Arteriovenous fistula	987 (67.6)	108 (39.3)	879 (73.3)	
Arteriovenous graft	94 (6.4)	15 (5.5)	79 (6.6)	
Central venous catheter	289 (19.8)	139 (51.3)	150 (12.6)	
Peritoneal catheter	86 (5.9)	8 (2.9)	78 (6.5)	
Other	19 (1.3)	5 (1.8)	14 (1.2)	
Oral medication, *n* (%)				
ARB	850 (57.7)	98 (35.6)	752 (62.8)	< 0.001
ACE inhibitors	131 (8.9)	16 (5.8)	115 (9.6)	0.05
β-Blockers	519 (35.2)	80 (29.1)	439 (36.6)	0.02
Calcium antagonist	1178 (79.9)	184 (66.9)	994 (82.8)	< 0.001
Aldosterone antagonist	75 (5.1)	13 (4.7)	62 (5.2)	0.8
Statins	596 (40.4)	76 (27.6)	520 (43.3)	< 0.001
Loop diuretics	979 (66.4)	144 (52.4)	835 (69.6)	< 0.001
Vitamin D receptor activator	405 (27.5)	40 (14.5)	365 (30.2)	< 0.001
Antiplatelet agents	450 (30.5)	68 (24.7)	382 (31.8)	0.02
Conditions at first dialysis session				
Presence of heart failure symptoms, *n* (%)	452 (30.7)	114 (41.5)	338 (28.3)	< 0.001
Predialysis body weight (kg), mean ± SD	60.2 ± 13.8	58.7 ± 14.8	60.5 ± 13.6	0.05
Predialysis systolic blood pressure (mmHg), mean ± SD	151 ± 26	148 ± 28	152 ± 25	0.03
Predialysis diastolic blood pressure (mmHg), mean ± SD	77 ± 15	76 ± 18	77 ± 14	0.5

*eGFR* estimated glomerular filtration rate, *ARB* angiotensin-receptor blocker, *ACE* angiotensin-converting enzyme, *SD* standard deviation

Echocardiography was performed in 270 (90.9%) of late referral patients and 966 (80.5%) in absence of late referral patients. The median ejection fraction was 61% (interquartile range; 50.0%–67.3%) in late referral patients and 63% (interquartile range; 53.0%–69.0%) in absence of late referral patients. In our study, enrolled patients without echocardiographic information were younger and characterized by lower CVD risks (Additional file 1: Table S1).

### Mortality

During a median follow-up period of 2.2 years, 260 all-cause deaths occurred, 99 of which were CVD-related deaths. The Cox proportional hazards models (Table 2) showed that late referral was associated with all-cause mortality after adjustment for demographics and comorbidities (model 2: adjusted hazard ratio [HR], 1.47; 95% confidence interval [CI], 1.10–1.96). Model 3 adjusted for model 2 plus malnutrition and bone mineral metabolism was as follows: adjusted HR 1.35; 95% CI, 1.00 to 1.82. Finally, model 4 adjusted for model 3 plus anemia was as follows: HR 1.33; 95% CI, 0.99 to 1.79. We also found that a history of volume overload was associated with increased mortality after adjustment for demographics, comorbidities, malnutrition, and bone mineral metabolism (model 3: adjusted HR, 1.39; 95% CI, 1.06–1.81).

**Table 2:** Multilevel Cox proportional hazards models for late nephrologist referrals and clinical factors of all-cause mortality

Factors of all-cause mortality (n = 1475)	Model 1^a^	Model 2^b^	Model 3^c^	Model 4^d^
	AHR (95% CI)	AHR (95% CI)	AHR (95% CI)	AHR (95% CI)
Late referral to a nephrologist	**1.41 (1.06–1.89)**	**1.47 (1.10–1.96)**	**1.35 (1.00–1.82)**	1.33 (0.99–1.79)
Age (per year)	**1.05 (1.04–1.07)**	**1.05 (1.04–1.06)**	**1.05 (1.04–1.06)**	**1.05 (1.03–1.06)**
Sex (male)	**1.61 (1.21–2.13)**	**1.49 (1.12–1.98)**	**1.46 (1.09–1.94)**	**1.47 (1.10–1.77)**
Medical history				
Volume overload	**1.53 (1.18–1.97)**	**1.39 (1.06–1.81)**	**1.39 (1.06–1.81)**	**1.35 (1.03–1.77)**
Malignancy		**1.59 (1.15–2.20)**	**1.58 (1.14–2.20)**	**1.53 (1.11–2.13)**
Comorbidity				
Diabetes mellitus		0.98 (0.76–1.27)	0.96 (0.75–1.24)	0.99 (0.76–1.28)
Atherosclerotic disease		**1.72 (1.32–2.22)**	**1.68 (1.30–2.18)**	**1.69 (1.30–2.19)**
Laboratory data				
Serum albumin (g/dL)			**0.72 (0.58–0.89)**	**0.76 (0.61–0.95)**
Phosphate (mg/dL)			1.04 (0.97–1.11)	1.03 (0.96–1.10)
Hemoglobin (g/dL)				**0.92 (0.84–1.00)**

*AHR* adjusted hazard ratio, *CI* confidence interval

^a^Adjusted for age, sex, and history of volume overload

^b^Adjusted for variables in model 1 plus history of malignancy and comorbidities of diabetes mellitus and atherosclerotic disease

^c^Adjusted for variables in model 2 plus serum albumin and phosphate

^d^Adjusted for variables in model 3 plus hemoglobin

Bold data is one that does not include the value 1 in the 95% confidence interval

### Effects and the relationship between late referral and history of volume overload on prognosis

**Fig. 2:**
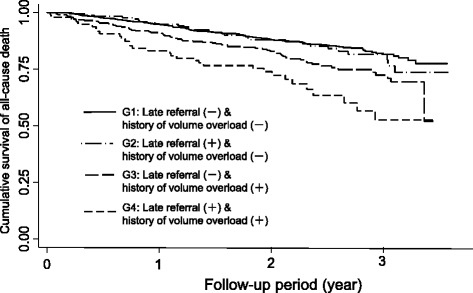
The overall survival of all-cause mortality among the four groups stratified according to presence or absence of late referral and with or without history of volume overload (G, group)

**Fig. 3:**
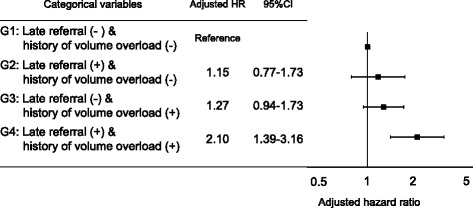
The relationship between late referral and history of volume overload on the all-cause mortality using Cox proportional hazards models adjusted for age, sex, diabetes mellitus, atherosclerotic disease, and malignancy

To investigate the influence of history of volume overload on prognosis in terms of referral timing to a nephrologist, the 1475 patients enrolled were stratified into four groups (G), from G1 to G4, according to the timing of referral and the with or without history of volume overload. The G1 group had the lowest risk of death with absence of late referral and without history of volume overload; G2 had late referral without history of volume overload; G3 had absence of late referral with a history of volume overload; and G4 had the highest risk of death with late referral with a history of volume overload. Kaplan-Meier curves for the four all-cause mortality groups are shown in Fig. 2. Subsequently, we evaluated the effect of the relationship between late referral and history of volume overload on the all-cause mortality using a multivariate Cox proportional hazards models (Fig. 3). When compared to the absence of late referral and no history of volume overload (G1), we found that patients with both late referral and a history of volume overload (G4) had a significantly higher risk of all-cause mortality after adjusting for age, sex, diabetes, atherosclerotic disease, albumin, and phosphate. Significant interaction was not observed between late referral and history of volume overload, but fully adjusted HRs from G2 to G4 compared with G1 were as follows: G2 HR, 1.15; 95% CI, 0.77 to 1.73; G3 HR, 1.27; 95% CI, 0.94 to 1.73; and G4 HR, 2.10; 95% CI, 1.39 to 3.16.

We also surveyed the detailed etiology of deaths in each group (Table 3). Among groups G1 to G4, the incidence rate of all-cause death was the highest at 17.6 per 100 persons-year in the G4 group (late referral with history of volume overload). We also assessed the effects on CVD-related mortality using competing risk analysis. In this analysis, the effect of late referral on CVD-related death was unclear (Additional file 2: Figure S1). The “other” etiologies of death included a clinically diagnosed case of natural death due to aging, withdrawal of dialysis, and death due to suffocation. In the G2 group (late referral with no history of volume overload), the proportion of CVD-related death was the lowest, and the proportion of “other” causes of death was the highest.

**Table 3:** All-cause mortality rates and causes of death among the four groups stratified by referral timing and history of volume overload

	G1: Not late referral without history of volume overload (*n* = 916)				G2: Late referral without history of volume overload (*n* = 178)				G3: Not late referral with history of volume overload (*n* = 284)				G4: Late referral with history of volume overload (*n* = 97)			
Total number of all-cause deaths, *n*	133				29				66				32			
Incidence rate of all-cause death, *per 100 person-years*	6.5				7.2				10.7				17.6			
Cause of death																
Cardiovascular disease, *n* (%)	45	(34%)			5	(17%)			30	(45%)			19	(57%)		
Heart failure			10	(8%)			2	(7%)			14	(21%)			9	(27%)
Coronary heart disease			5	(4%)			0	(0%)			7	(11%)			5	(15%)
Sudden cardiac death			14	(11%)			1	(3%)			4	(6%)			1	(3%)
Stroke			13	(10%)			2	(7%)			3	(5%)			4	(12%)
Aortic disease			3	(2%)			0	(0%)			2	(3%)			0	(0%)
Infection, *n* (%)	33	(25%)			6	(21%)			12	(18%)			3	(9%)		
Malignant tumor, *n* (%)	32	(24%)			6	(21%)			5	(8%)			2	(6%)		
Other, *n* (%)	14	(11%)			10	(34%)			15	(23%)			5	(15%)		
Unknown, *n* (%)	9	(7%)			2	(7%)			4	(6%)			3	(9%)		

*G* group

## Discussion

Our multicenter prospective cohort study that examined various clinical conditions and outcomes in detail showed that both late referral to a nephrologist and history of volume overload were associated with an increased risk of all-cause mortality after adjusting for age, sex, comorbidities, and laboratory values. The additional analysis of the relationship between history of volume overload and late referral stratified patients into four groups based on combinations of the presence or absence of these factors with the data inserted into Cox proportional hazards models, showed that the late referral coexisting was associated with a history of volume overload increased mortality after initiating dialysis.

Several studies have found that late referral to a nephrologist is associated with higher mortality and hospitalization [1, 22–24]. The results of the present study were consistent with previous estimates of the effects of late referral on mortality. In addition, the Cox proportional hazards models in our study showed that the effects of late referral on overall death were attenuated when adjusted for modifiable factors that can be managed by a nephrologist in pre-ESRD. These results suggest that the lack of patient management by renal specialists in pre-ESRD may potentially affect the survival prognosis after initiating dialysis. Thus, even in Japanese patients newly initiating dialysis who were elderly and exhibited multiple comorbidities, we demonstrated that late referral to a nephrologist was harmful. Clinically, of course, the idea is reasonable that a 3-month attention by a nephrologist is insufficient to optimize all complications derived from advanced renal failure. Therefore, this is considered the minimum period necessary to prepare for RRT (e.g., choice of dialysis modality, creation of dialysis access). Generally on dialysis induction, the patient with late referral often presents in a condition that is clinically challenging, such as pulmonary congestion or hyperkalemia, and is urged to prepare for dialysis access and initiate RRT. Nevertheless, late referral is not only the challenging situation, late referral patients usually also present with concurrent clinical, hematological, hormonal, and metabolic abnormalities, such as anemia, malnutrition, hyperparathyroidism, hyperphosphatemia, hypocalcaemia, hypertension, and congestive heart failure, all of which could linked to poor dialysis outcomes [25]. As in previous studies, late referral patients in our cohort had fewer prescriptions for angiotensin-receptor blockers, angiotensin-converting enzyme inhibitors, vitamin D receptor activators, and tended to start the first session of maintenance dialysis with central venous catheters. However, our study showed that late referral also adversely affects prognosis after adjustment for the patient’s underlying level of clinical conditions. Thus, the effect of late referral was not simply that medical intervention was insufficient, but it also resulted in impaired multidisciplinary renal treatment during progression from CKD to ESRD and compromised dialysis preparation by renal specialists.

We also focused on history of volume overload in patients with a late referral. In our study, a clinical history of volume overload was significantly associated with late referral, and patients with a late referral presented a higher proportion of heart failure symptoms at the first dialysis session. Since concerns have been raised about the relationship between the history of volume overload and late referral, we investigated the interaction between history of volume overload and late referral. Although significant interaction was not demonstrated, we found that the coexistence of late referral and history of volume overload had a high risk of death after dialysis. This effect was greater than expected given the associations of volume overload and late referral alone. In other words, if patients with a previous history of volume overload were referred to a nephrologist at the later phase of stage 5 CKD, their survival prognosis may decrease. Recent studies, whereby volume overload was objectively measured using a bioelectric impedance method, reported that volume overload was associated with a rapid decline in eGFR and increased mortality risk in advanced CKD patients [11, 26]. In another study using the bioimpedance method, among the 338 patients with stage 3–5 CKD, only 48% were euvolemic, and approximately 20% of patients with volume overload were occult volume overload [14]. It is difficult to use the bioimpedance method universally to identify occult volume overloads, but a clinically identified past history of volume overload may suggest the existence of potential volume overload. Excess volume status could lead to activation of malnutrition and inflammation [14], and may influence vascular and endothelial function leading to arterial stiffness, atherosclerosis, and left ventricular hypertrophy [27]. In our cohort, approximately half of the patients with history of volume overload died due to CVD. Therefore, if CKD patients with history of volume overload experience a delay in referring to a nephrologist, the prognosis would be worsened due to the lack of optimal management of complications from advanced CKD and not being able to achieve a better placement of dialysis access such as arteriovenous fistula, both associated with adverse outcome. We suggest, based on real world evidence, that a clinical history of volume overload has a potential negative impact on patients with advanced CKD. If a non-renal specialist manages advanced CKD patients with a history of volume overload, dietary salt restriction, managing volume excess, and timely referral to a nephrologist are important to improve the patient’s outcome.

This study has several limitations. First, the onset and severity of clinical history and comorbidities including a history of volume overload is unknown. The history of volume overload was determined before dialysis induction. We did not use a bioimpedance method that could evaluate the presence of volume overload objectively and strictly. We defined volume overload using the clinically convenient indicators of physical findings and chest X-rays. Compared to a previous study using the bioimpedance method, the severity of volume overload defined in our study may be severely biased. Second, echocardiography only measured left ventricular ejection fraction; left ventricular hypertrophy and left ventricular mass were not measured. In addition, as the timing of echocardiography includes the phase of dialysis induction, the ejection fraction may have been modified by asymptomatic heart failure developed just before the dialysis initiation. Third, there are several potential selection biases in the present study. We did not consider the patients who died during hospitalization at the initiation of dialysis or those who died before reaching ESRD. A lead-time bias may also exist because there were no definite criteria for starting maintenance dialysis. The timing of dialysis initiation was determined according to the subjective judgment of the attending physician. In patients with a high risk of death dialysis can be initiated earlier. This, may provide a positive lead-time bias for late referral patients and may attenuate the association between late referral and time-to-death. Fourth, some potentially important residual confounders were not measured, including longitudinal data about the quality of dialysis which may be more important and relevant to the outcome.

## Conclusions

In conclusion, late referral and history of volume overload were identified as risk factors for overall mortality after initiating dialysis. Our findings also suggest that delayed referral to a nephrologist potentially had a negative influence on the survival prognosis of CKD patients with a history of volume overload. To avoid late referral, further studies are needed to determine the optimal timing for referral while taking the patient’s clinical conditions and healthcare resources into consideration. An improved understanding of the decision-making processes of non-renal specialists regarding timely referral to a nephrologist will enhance high-risk CKD patient care.

## Additional files

Additional file 1: **Table S1.** Background characteristics of patients who underwent or did not undergo echocardiography. (DOCX 35 kb)

Additional file 2: **Figure S1.** Cumulative incidence functions for CVD-related death for four groups stratified according to late nephrologist referral and history of volume overload. (PDF 21 kb)

## Abbreviations

AICOPP: Aichi Cohort Study of Prognosis in Patients Newly Initiated into Dialysis
CKD: Chronic kidney disease
CVD: Cardiovascular disease
eGFR: Estimated glomerular filtration rate
ESRD: End-stage renal disease
RRT: Renal replacement therapy
