# Supplementary material for: Unfavorable effects of history of volume overload and late referral to a nephrologist on mortality in patients initiating dialysis: a multicenter prospective cohort study in Japan
Source: BMC Nephrol. 2018 Mar 14;19:65. doi: 10.1186/s12882-018-0859-8 (PMC5853026; doi:10.1186/s12882-018-0859-8)
Supplement: Supplementary file 1 — Table S1. Background characteristics of patients who underwent or did not undergo echocardiography. (DOCX 35 kb) [file 12882_2018_859_MOESM1_ESM.docx]

**Table S1.** Background characteristics of patients who underwent or did not undergo echocardiography

|  | Performed echocardiography  (*n* = 1216) | Not performed echocardiography  (*n* = 259) | *P* value |
| --- | --- | --- | --- |
| Characteristics |  |  |  |
| Age (years), mean ± SD | 67.9 ± 12.8 | 65.6 ± 14.2 | 0.02 |
| Male sex, *n* (%) | 818 (67.3) | 187 (72.2) | 0.1 |
| Medical history, *n* (%) |  |  |  |
| Volume overload | 337 (27.7) | 44 (17.0) | <0.001 |
| Admission for heart failure | 269 (22.1) | 39 (15.1) | 0.01 |
| Amputation | 24 (2.0) | 0 (0) | 0.02 |
| Malignancy | 126 (10.4) | 32 (12.4) | 0.3 |
| Comorbidity, *n* (%) |  |  |  |
| Diabetes mellitus | 656 (54.0) | 111 (42.9) | 0.001 |
| Atherosclerotic disease | 337 (27.7) | 68 (26.3) | 0.6 |
| Coronary heart disease | 218 (17.9) | 33 (12.8) | 0.05 |
| Valvular heart disease | 88 (7.2) | 11 (4.3) | 0.08 |
| Aortic disease | 69 (5.7) | 14 (5.4) | 0.9 |
| Peripheral artery disease | 67 (5.5) | 9 (3.5) | 0.2 |
| Cerebrovascular disease | 106 (8.7) | 30 (10.6) | 0.1 |
| Chronic obstructive pulmonary disease | 40 (3.3) | 10 (3.9) | 0.6 |
| Peptic ulcer disease | 48 (4.0) | 3 (1.2) | 0.03 |
| Dementia | 123 (10.1) | 26 (10.0) | 0.9 |
| Charlson comorbidity index | 4.9 ± 1.9 | 4.5 ± 1.7 | <0.001 |
| Laboratory values |  |  |  |
| Hemoglobin (g/dL), mean ± SD | 9.4 ± 1.5 | 9.2 ± 1.6 | 0.1 |
| Serum albumin (g/dL), mean ± SD | 3.0 ± 0.6 | 3.3 ± 0.6 | 0.01 |
| eGFR (mL/min per 1.73 m^2^), mean ± SD | 5.5 ± 2.3 | 5.1 ± 1.9 | 0.003 |
| Adjusted calcium (mg/dL), mean ± SD | 8.7 ± 1.0 | 8.3 ± 1.1 | <0.001 |
| Phosphate (mg/dL), mean ± SD | 6.3 ± 1.9 | 6.6 ± 1.9 | 0.04 |

*eGFR* estimated glomerular filtration rate, *SD* standard deviation
