# Supplementary figures and images for: Unfavorable effects of history of volume overload and late referral to a nephrologist on mortality in patients initiating dialysis: a multicenter prospective cohort study in Japan
Source: BMC Nephrol. 2018 Mar 14;19:65. doi: 10.1186/s12882-018-0859-8 (PMC5853026; doi:10.1186/s12882-018-0859-8)

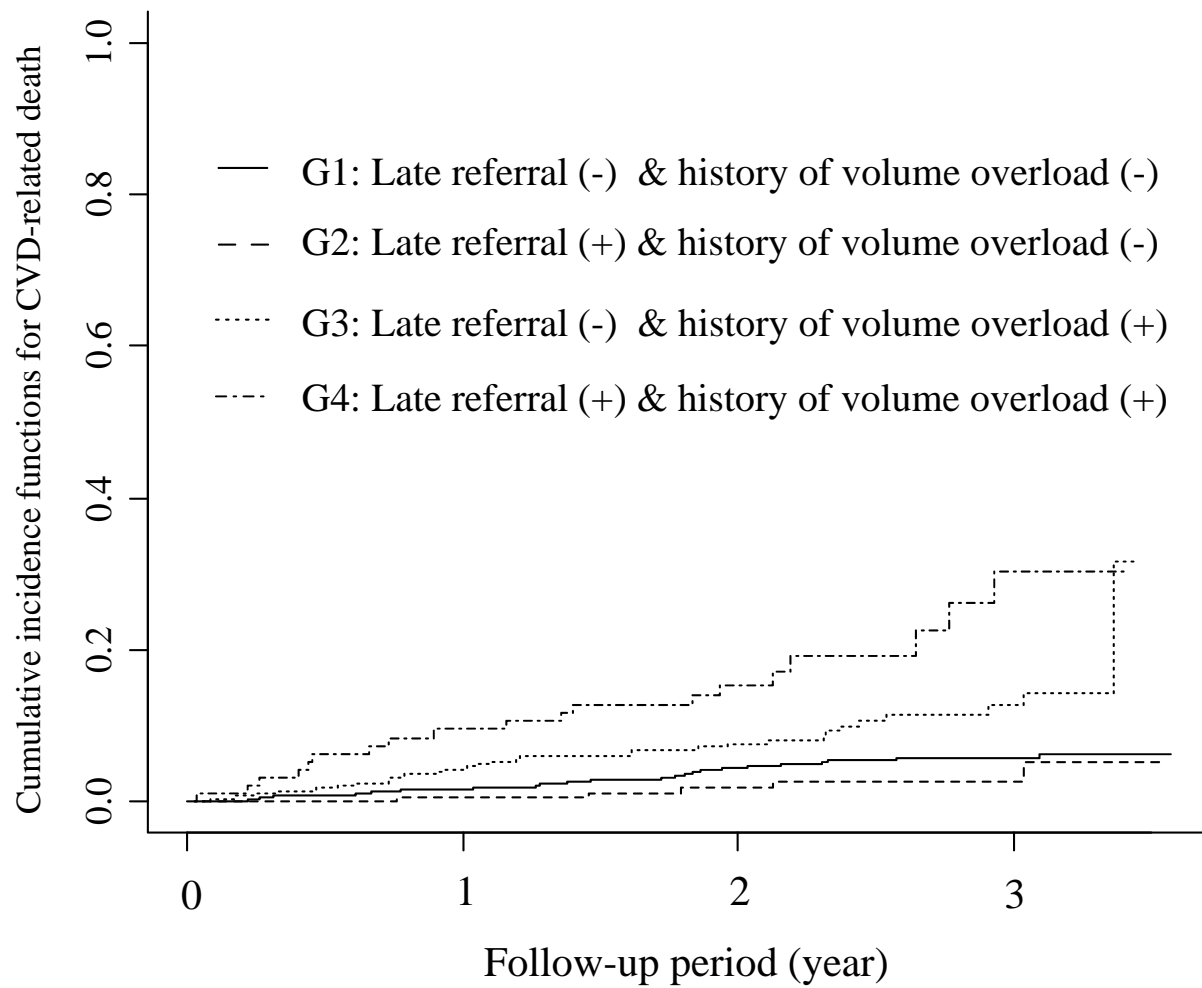

Supplement: Supplementary file 2 — Figure S1. Cumulative incidence functions for CVD-related death for four groups stratified according to late nephrologist referral and history of volume overload. (PDF 21 kb) [file 12882_2018_859_MOESM2_ESM.pdf]
